# Current status and upcoming developments for online adaptive proton therapy enabling a closed feedback loop for near real-time adaptation

**DOI:** 10.3389/fonc.2025.1660605

**Published:** 2025-12-17

**Authors:** Virginia Gambetta, Kristin Stützer, Christian Richter

**Affiliations:** 1 OncoRay – National Center for Radiation Research in Oncology, Faculty of Medicine and University Hospital Carl Gustav Carus, TUD Dresden University of Technology, Helmholtz-Zentrum Dresden-Rossendorf, Dresden, Germany; 2 Helmholtz-Zentrum Dresden-Rossendorf, Institute of Radiooncology – OncoRay, Dresden, Germany; 3 Department of Radiotherapy and Radiation Oncology, Faculty of Medicine and University Hospital Carl Gustav Carus, TUD Dresden University of Technology, Dresden, Germany; 4 German Cancer Consortium (DKTK), Partner Site Dresden, and German Cancer Research Center (DKFZ), Heidelberg, Germany

**Keywords:** cancer, proton therapy, online adaptive proton therapy, near real-time adaptation, volumetric imaging, online plan optimization, online QA, treatment verification

## Abstract

Proton therapy (PT) has the potential to deliver conformal doses to the tumor while sparing normal tissue, but is highly susceptible to treatment uncertainties. The occurrence of anatomical changes during PT treatments has a major impact on the delivered dose, often necessitating plan adaptations that are typically performed offline and require a few days before the adapted plan is ready. In order to react promptly to detected anatomical changes, online adaptive proton therapy (OAPT) has been proposed with the goal of adapting the plan while the patient is on the treatment couch. First OAPT workflows for daily plan adaptation that are effective against interfractional anatomical variations have reached clinical application. However, even faster OAPT workflows are needed to cope with faster anatomical changes. Near real-time adaptive PT (NAPT) relying on online *in vivo* treatment verification can be a potential solution for many tumor entities (e.g., thoraco-abdominal tumors), which would greatly benefit from the conformality of PT, but are presently challenging to treat with proton beams due to the influence of intrafractional variations. In addition, NAPT offers the opportunity to achieve the long-awaited closed PT feedback loop. In this paper we review the required tasks and necessary components in an OAPT workflow for the application of near real-time adaptation, proceeding sequentially from volumetric imaging for online plan adaptation up to online verification during delivery. Available technology and upcoming developments are discussed. Several aspects regarding regulatory approval, cost-benefit related issues and additional beyond-the-loop tasks are also addressed.

## 1 Introduction

Due to the characteristic behavior of proton beams and their energy release in tissues, proton therapy (PT) is particularly sensitive to anatomy variations occurring during the course of the treatment (1). A modification of the patient anatomy may result in underdosage to the tumor, unnecessary high doses to the normal tissue or even overdosage to the organs-at-risk (OAR), potentially leading to severe side effects. PT remains constrained for certain entities (2), particularly in the abdominal region. In parallel with the advent of latest technological developments, new radiotherapy (RT) treatment modalities have emerged that introduce an adaptive approach. The concept of adaptive radiotherapy (ART) based on a systematic feedback loop is well-known (3) and can be classified into three main time regimes: offline (adaptation performed between fractions), online (adaptation performed directly prior to delivery, while the patient is on the treatment couch) and real-time (adaptation performed within fraction delivery) (4). Online ART has become a reality in photon-based radiotherapy (XRT) with the availability of hybrid systems of imaging plus linear accelerator (LINAC), like magnetic resonance (MR)-LINACs (5), tomotherapy (6) and systems based on cone-beam computed tomography (CBCT) (7). Within ART, positron emission tomography (PET)-integrated systems have also been recently proposed (8) that target biological changes in the tumor rather than anatomical variations, thereby propelling the concept of biology-driven adaptation (9).

**Figure 1:**
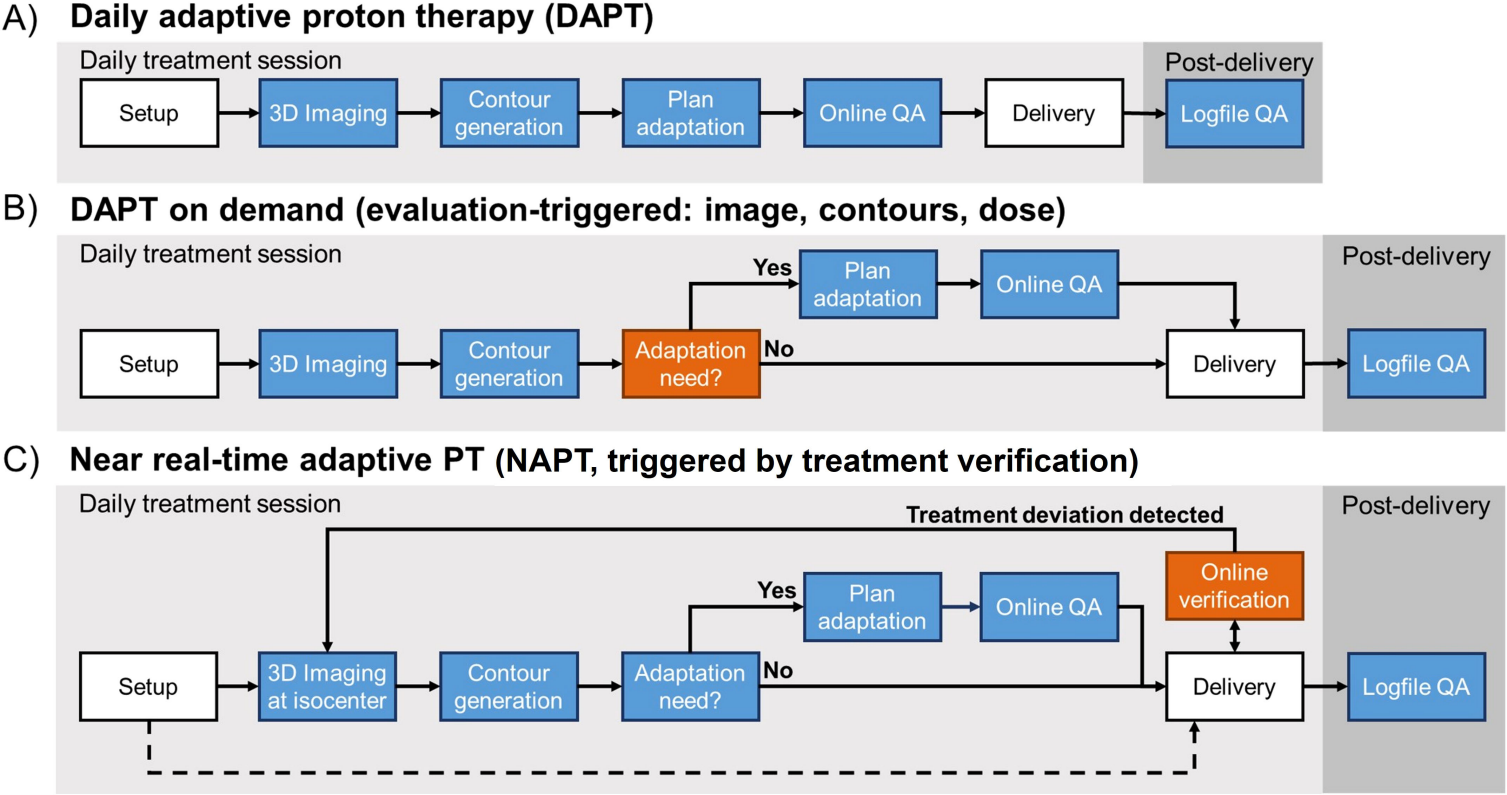
Different workflows representing the multifaceted OAPT landscape, including daily and post-delivery tasks. Standard PT tasks (in white) are differentiated from major OAPT components (in blue). Workflow-specific OAPT components are highlighted in orange. Note that, while integrated CBCT systems are now widely established for patient setup in PT, in the context of OAPT an in-room 3D imaging system capable of providing a high-quality volumetric image for planning is essential and is crucial for near real-time applications. In standard DAPT **(A)** the online adaptation follows volumetric imaging and online contouring tasks. To reduce the daily burden, daily adaptation can be performed on demand **(B)** only after evaluation of the actual adaptation need. Finally, NAPT **(C)** relies on treatment verification in parallel with delivery to account for unexpected intrafractional anatomical variations and trigger the image acquisition and eventual adaptation only after a treatment deviation is detected. This differs from standard **(A)** or evaluation-triggered **(B)** DAPT, where the adaptation happens before delivery. If no treatment deviation is identified, the clinical workflow can proceed from setup directly to delivery as in standard clinical practice. Real-time adjustments involving motion management techniques are not represented. Treatment verification without triggering capability may be used to independently monitor the delivery also in workflows **(A, B)**.

Robust optimization in PT, as implemented in commercial treatment planning systems (TPS) today, accounts only for positional shifts within an otherwise unchanged anatomy. However, anatomical changes, such as variations in cavity filling or tumor size, can lead to clinically significant differences. Given the large impact of anatomical variations on the conformity of the proton dose distribution, the spread of online ART has sparked interest for potential applications in PT (10, 11), with many PT centers actively working towards clinical implementation of this novel approach (12–14). The concept of online adaptive PT (OAPT) is not yet as established as its photon counterpart and refers generally to the opportunity of performing a plan adaptation (and related tasks) while the patient is on the treatment couch during the daily treatment session. This review focuses on online adaptations due to anatomical changes. There are different flavors of OAPT (**Figure 1**). They depend on the one hand on the timing of the addressed anatomical changes (and the necessity of potential plan adaptations per treatment and fraction) and on the other hand on the time needed to perform the adaptation itself (15).

Interfractional changes like weight changes, tumor shrinkages or growth, relevant changes in cavity filling, and substantially different fill levels of the digestive organs with related shifts of nearby soft tissue happen on a timescale of hours or days (16). These variations can be dealt with daily adaptive PT (DAPT), with the goal of adapting the plan to the daily anatomy. In a standard DAPT workflow (**Figure 1A**), the online plan adaptation follows the conventional setup and 3D imaging. This way the treatment plan is adapted to the current daily anatomy based on an image acquired prior the delivery. Attempts at implementing such a workflow have been recently proposed (12) and first clinical realizations have been reported (17, 18).

Generating the online adapted plan at each fraction places an undeniable burden on patient comfort and daily workload. To reduce the daily burden, an evaluation-based DAPT “on demand” is envisaged (**Figure 1B**), where the online adaptation is possibly performed after an assessment of its need based on the daily acquired 3D image and/or projected dose distribution or even on the information from pre-delivery range assessment like range probing (19). If there is no need for adaptation, the workflow can continue with the delivery of the initial treatment plan.

However, in order to address faster changes in organ position and deformation, an even faster action is required that is capable of detecting and correcting for intrafractional variations. Such anatomical changes can happen on timescales of minutes (like urinary activity, organ drift), or seconds (like peristalsis, respiratory movements, cardiac motion) (20–22). In particular, respiratory movements are usually handled prospectively via techniques of motion management, like suppression of motion, 4D imaging and optimization, continuous motion surrogate monitoring, and appropriate changes in the beam delivery sequence (gating, repainting), etc. (15, 16). Motion management alone, however, is not able to compensate for the entire intrafractional range of movements, especially when dealing with irregular and/or unexpected variations, highlighting the need for a near real-time adaptive approach that includes online generation of an adapted plan (2, 23). For example, entities like thoraco-abdominal (e.g., lung, esophageal, liver, pancreas), gynecological and genitourinary tumors are greatly influenced by unexpected intrafractional variations and located in close position to gastrointestinal, cardiac, pulmonary and/or urinary OAR (2, 16), thus requiring steep dose distributions. For such entities a shift towards near real-time adaptation has already been observed in XRT after the advent of MR-guided radiotherapy (24–26). This emphasizes the necessity of adopting similar approaches for PT, which can offer superior conformality but is presently limited. With near real-time adaptation in PT, analogous to XRT, it would be possible to continuously monitor treatment delivery and promptly react to irregular intrafraction anatomical changes as they occur. This differs from standard or evaluation-triggered DAPT, in which the adaptation is only performed before the treatment delivery. Near real-time adaptive PT (NAPT) (**Figure 1C**) can address this issue by adapting the treatment plan using information on treatment deviations from online treatment verification systems that run concurrently with delivery. Upon identification of a deviation between the delivered and the expected proton dose, an online adaptive workflow is initiated with the acquisition of a 3D image and the assessment of the adaptation need. On the one hand, this approach would allow a rapid response to unforeseen anatomical changes and, on the other hand, it would provide a safety net to ensure that the planned dose is delivered as indicated and that further plan adaptation is only applied when necessary. This NAPT workflow, which involves imaging, contour generation, plan adaptation, and approval under stricter time constraints, is not yet available in a truly real-time setting due to the time currently required to complete these critical operations before resuming delivery.

In this sense, at the time of this review an actual “real-time” adaptive PT can only be represented by operations of motion management happening during the daily treatment session that do not require a beam delivery interruption or a treatment plan modification, leading instead to adjustments of the beam geometry or delivery sequence (15, 27, 28). These real-time operations have also been previously referred to as an in-line adaptive workflow (11).

This review outlines step-by-step the necessary components within a daily treatment session to implement a NAPT closed feedback loop after standard patient setup, describing the current research status and highlighting areas that require further investigation. Each major step in the workflow (volumetric imaging for online treatment planning, online contour generation, decision on online adaptation need, online plan adaptation – optimization strategy and dose calculation, pre-delivery quality assurance of the online adapted plan, online treatment verification) will be discussed in dedicated sections. Additional aspects regarding integration, regulatory approval and tasks that are performed outside the loop will also be addressed.

## 2 Volumetric imaging for online treatment planning

The availability of 3D imaging in the treatment room, preferably keeping the patient in treatment position, is essential for identifying anatomical changes in the timeframe for OAPT. Given the stricter timeframe and potential for adaptation during paused/suspended delivery, 3D imaging in treatment position is especially relevant for in NAPT. The quality of the acquired images must be adequate for the online plan adaptation of proton-based treatments, i.e., it must ensure the correct retrieval of the stopping power ratio (SPR) for accurate range and dose calculation. Image-guided XRT has propelled the development of volumetric image guidance tools (29), many of which are now being investigated for OAPT. Here pre-delivery imaging techniques based on in-room (fan-beam) CT, CBCT, MR imaging (MRI) and proton CT are discussed in terms of technological advancement, the ability to retrieve the current 3D patient’s anatomy and related SPR map for proton dose calculation, and the suitability for near real-time adaptation (**Table 1**). In-room imaging tools providing additional information for real-time motion management are briefly mentioned.

**Table 1:** Overview of the main in-room pre-delivery volumetric imaging systems depicting their properties and suitability for online (near real-time) plan adaptation in PT.

Imaging technique	SPR prediction	Imaging in treatment position (at isocenter)	Continuous imaging during delivery	Current application in PT	Strengths (+)	Limitations (-)
In-room (fan-beam) CT	Clinical gold standard, direct SPR prediction with spectral information (DECT or photon counting CT)	No for CT on rails; Yes for upright PT systems	No	For daily positioning and routine control CTs acquisition in clinical treatments; use as volumetric imaging for online adaptation in first clinical DAPT application has been reported (17)	+ Established technique + High geometrical accuracy + Accurate quantitative information (CT number accuracy) + Low-dose imaging protocols available for online applications + 4D-CT available + Used in first clinical DAPT applications	− Imaging not in treatment position − Potentially complex integration into the workflow − Time required to move patient and CT system − No real-time monitoring during treatment
CBCT	Correction techniques required to overcome image artefacts	Yes, several configurations available: nozzle-mounted, gantry-mounted, ceiling-mounted, couch-mounted (29)	No	For patient set up and daily imaging in clinical treatments	+ Imaging in treatment position + Already available for online adaptive XRT + Several vendor/configuration options + 4D-CBCT available	− Several image quality issues (field of view, CT number accuracy) currently limit its usage for proton treatment planning (not yet clinical) − No real-time monitoring
MRI	Conversion to CT numbers or SPR required	Potentially yes, in the foreseen in-room/integrated configuration for fixed beamlines (30, 31);	Potentially yes (foreseen for fixed beamline configurations); not currently available	Preclinical research and first clinical applications with shuttle-based systems (32, 33)	+ No additional ionizing radiation + Enhanced resolution for soft tissue + Better delineation of tumor due to higher contrast + Potential real-time monitoring + First clinical experiences available (shuttle-based systems)	− Integration in the treatment room limited to fixed beamlines (not compatible with gantry) − Mutual interference of proton beam and magnetic field − Conversion of MR data to CT numbers for treatment planning required (additional uncertainties) − Inferior geometrical accuracy (distortion)
Proton CT	Direct SPR information	Yes, relying on the same system used for treatment delivery	In principle possible (mixed beam with different energies)	Preclinical research (34, 35)	+ Direct retrieval of SPR	− Degraded information due to multiple Coulomb scattering − Limited to very few entities due to geometrical conditions (distance between detectors before and after patient) − Not available as medical product

**Figure 2:**
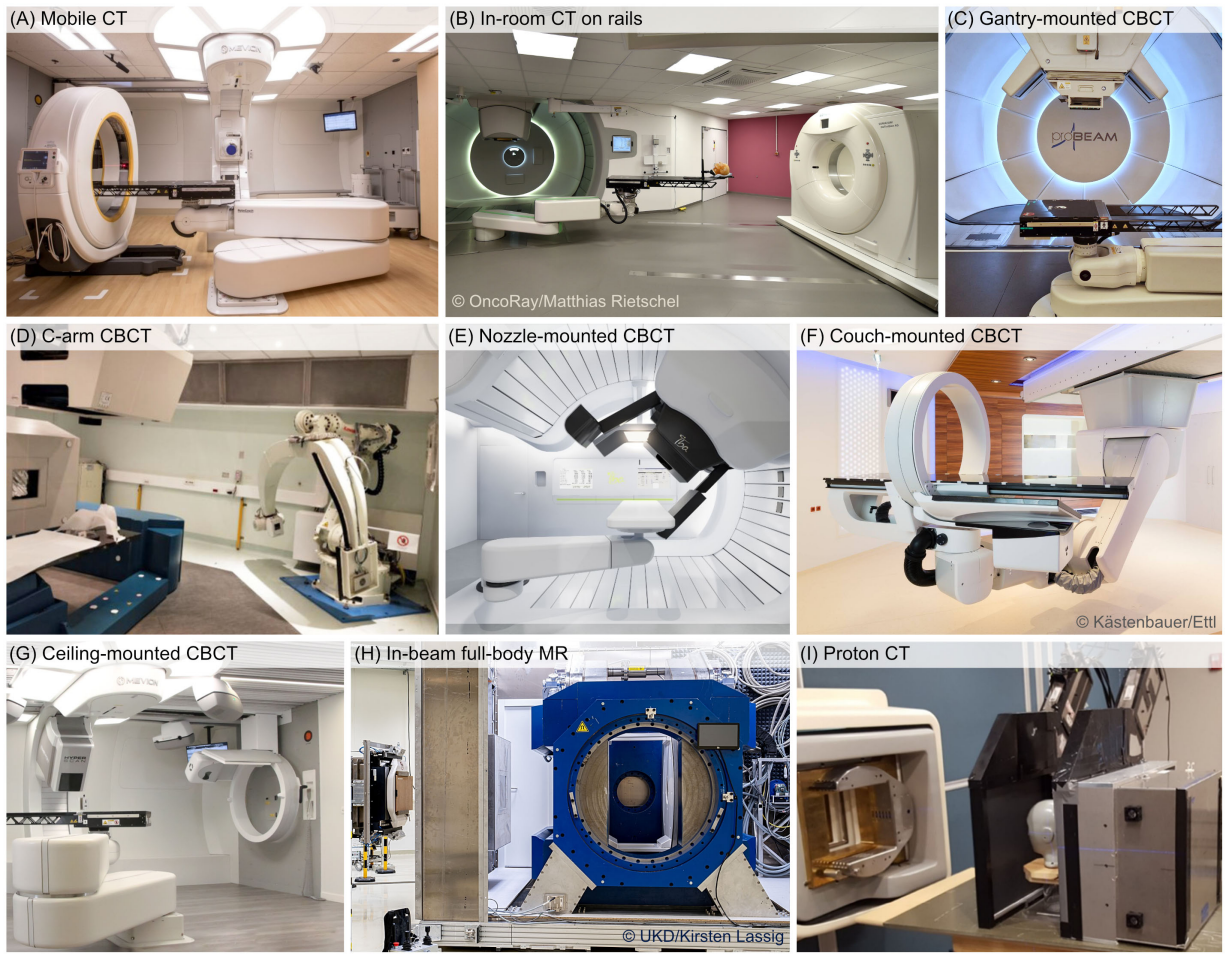
Overview of discussed volumetric imaging solution for treatment planning: **(A)** mobile CT scanner (reproduced from https://www.itnonline.com/content/orlando-health-and-mevion-offer-advanced-diagnostic-image-guidance-proton-therapy with permission), **(B)** Siemens in-room CT on rails at OncoRay (reproduced from https://www.hzdr.de/db/Cms?pOid=43592&pNid=99 with permission), **(C)** Varian gantry-mounted CBCT at Paul Scherrer Institute, **(D)** CNAO C-arm mounted CBCT (reproduced from (36) under CC-BY 4.0 license), **(E)** IBA nozzle-mounted CBCT (reproduced from https://www.itnonline.com/content/iba-and-philips-step-commercial-collaboration-brazilian-proton-therapy-market with permission), **(F)** Medphoton (Brainlab) couch-mounted CBCT at MedAustron, **(G)** Medphoton (Brainlab) ceiling-mounted CBCT at Maastro Proton Therapy (courtesy of Mevion Medical Systems and Maastro Proton Therapy), **(H)** full-body, rotatable MR scanner integrated at horizontal beamline at OncoRay, (reproduced from https://www.hzdr.de/db/Cms?pOid=71010&pNid=0 with permission) **(I)** experimental proton CT prototype (reproduced from (35) under a CC-BY 4.0 license).

In-room (fan-beam) CT is a well-known pre-treatment imaging solution for checking the anatomy in treatment position and for setup and positioning. Developments of mobile CT scanners (**Figure 2A**) to perform in-room CT for OAPT have been observed (37, 38) and proposed for clinical use (39), although the integration in a fast and efficient workflow is challenging. CT scanners on rails have a fixed position in the room (**Figure 2B**). Despite the associated costs and impact on the overall available space in the treatment room, they are at present gaining traction for use in DAPT workflows (12, 17). In-room CT offers several advantages regarding image quality, the most accurate prediction of the SPR for PT planning including dual-energy CT (DECT) based SPR prediction (39, 40) and the option to perform 4D-imaging to monitor interfractional changes in the respiratory pattern (16), however, it is currently not optimal for near real-time adaptation. In-room CT scans are acquired distant from the actual delivery position and usually incapable of returning images matched to the isocenter. Before proceeding further with the adaptation, the in-room CT images must be first registered to the reference image (e.g., planning CT), in order to share the same reference frame. Moreover, the reference frame of the in-room CT and its position within the treatment room must be also correctly related with the room coordinate system. In order to correctly locate at room isocenter a specific point of the treatment volume identified in the CT scan, a precise and reproducible position of the robot for patient positioning (or patient positioning system) is required. Securing sufficient accuracy of the patient table position over the large distance from the PT gantry to the CT gantry is paramount. The integration of in-room CT systems with the delivery system still represents a challenge and is heavily facility-dependent (29). As a result, the insertion of such systems into a closed feedback loop for near real-time adaptation in PT is a complex undertaking. Still, in-room CTs are first choice for OAPT workflows as the fan-beam CT imaging is the gold-standard for accurate SPR prediction and dose calculation (39).

Over the past decade, CBCT has become available in newly built PT centers, with commercially available options from respective PT vendors seamlessly integrated into the treatment room. In detail, there are different configurations, like gantry-mounted (**Figure 2C**), mounted on robotic C-arms (**Figure 2D**), nozzle-mounted (**Figure 2E**), couch-mounted (**Figure 2F**), or ceiling-mounted (**Figure 2G**) (29). CBCT allows imaging at isocenter position and has thus received great interest for OAPT (18, 41). Its use in online adaptive XRT workflows is already established, with commercial systems such as Varian’s Ethos or Elekta’s C-arm LINACs (42–44), including novel developments of Elekta Evo and ONE (45), and the emerging implementation of 4D-CBCT enabling time-resolved imaging (46). However, the inherently low image quality of CBCT presents a significant challenge for its use in OAPT. Due to its technical design, CBCT is more susceptible to X-ray scattering within the patient or detector and beam hardening compared to fan-beam CT—especially in larger patients and at wide cone angles, such as in the upper abdomen or pelvis. Additional factors, including signal lag, limited detector efficiency, and field-of-view truncation, further degrade image quality. Since volumetric CBCT data is reconstructed from 2D projections acquired at different beam angles during a single gantry rotation (typically lasting 60 to 120 s) without patient couch movement, any patient motion can introduce additional artifacts. As a result, uncorrected CBCT images generally lack the image quality required to meet the strict CT number stability demands in PT for accurate dose calculation (47, 48). Hence, the adoption and clinically reliable validation of correction techniques is of utmost importance to allow for accurate proton dose calculations. Various correction methods to utilize CBCTs for dose computation in PT have been already investigated in several studies (49–52), and include deforming the planning (fan-beam) CT to the CBCT using deformable image registration (DIR), scatter correction methods, image correction utilizing deep convolution neural networks, histogram matching, and corrections via look-up tables. While CBCT offers an appealing option for near real-time adaptation at the isocenter (minimizing both the time and uncertainties associated with patient repositioning required for in-room CT), its routine clinical implementation for dose optimization remains unrealized despite extensive research efforts. As an initial step toward clinical translation, corrected CBCT datasets could be used exclusively for dose recalculations and as a trigger for acquiring a new fan-beam CT scan if clinically significant dosimetric changes are detected. With sufficient experience across a broad range of patient cases, direct plan adaptation based on CBCT with unified settings could then be introduced as a subsequent step. Moreover, CBCT integration into many commercial delivery and planning systems simplifies the integration into the OAPT closed feedback loop and gives the opportunity to perform prompt checks of the current anatomy, for example in case a treatment deviation is identified during delivery (e.g., by a complimentary treatment verification system). CBCT, however, like in-room CT, cannot be used to continuously monitor the patient anatomy and tumor location during beam delivery.

The CT-based solutions discussed so far assume that the patient is imaged and treated in recumbent position. Recently, interest in upright treatment and imaging has increased significantly (53). The idea of treating patients in a seated position is not new (54), but has gained renewed attention due to the development of commercial systems for vertical CT imaging together with reported clinical applications (55–58). The main driving factor towards the investigation of upright CT imaging and treatment is the opportunity of reducing the costs of the expensive PT facilities. In fact, employment of upright positioning would allow for a gantry-less treatment room, where the patient is rotated relative to the fixed beam instead of moving the beam around the patient with a gantry (59). An additional aspect to consider is that upright CT imaging offers in principle high flexibility and the opportunity of imaging at the isocenter. This is relevant for OAPT treatments, especially when aiming at faster time regimes. For certain tumor entities, treatment in an upright patient position may offer potential benefits and, in some cases, could even be more comfortable for patients. However, given the limited experience with this emerging technology – including specialized treatment chairs and volumetric imaging systems (59, 60) – several challenges remain. These must be addressed before definitive conclusions can be drawn regarding the treatment quality and clinical outcomes of upright PT. The positioning accuracy and reproducibility need to be quantified for different treatment sites. Upright PT will very much rely on fast online adaptation capabilities in case the positioning accuracy is inferior to supine treatments and most importantly clinical evidence of safe and efficient treatment needs to be generated (61, 62).

Finally, MRI is noteworthy for its ability to provide high-quality soft-tissue imaging without exposing patients to additional ionizing radiation from imaging. In XRT, MRI has already been integrated into daily adaptive workflows using MR-LINACs, where it enables continuous anatomical monitoring during treatment. Although MRI offers superior soft-tissue contrast compared to CT, it is inferior in terms of geometrical accuracy (distortions) and dose calculation applicability. The enhanced soft-tissue contrast may be relevant for NAPT and for challenging cases affected by significant intra-fractional variations (30, 63). However, MR-only dose calculation remains more complex in PT than in XRT, as accurate material characterization of bones and air from MR scans is crucial. As with CBCT, generating synthetic CT datasets from MR scans is being explored as a method for enabling SPR prediction, though it remains challenging. Various approaches, including bulk density overrides, anatomical atlases, voxel-based intensity assignments, DIR with CT datasets, and deep learning techniques, have been proposed to address this issue (31, 64). There are also significant challenges in integrating MRI into a PT treatment room: minimizing proton beam deflection in MR scanners (65) and ensuring the compatibility of dosimetry equipment with MRI (66, 67). Due to the impact of gantry rotation, which involves moving large amounts of steel, on the magnetic field of an MR scanner, integrating MRI into a gantry room seems practically impossible. As a result, translational research is currently focused on integrating MR scanners at horizontal beam lines. Since this could potentially be combined with an upright positioning, it may offer fewer limitations regarding the degrees of freedom for beam directions. Despite the challenges, research in MR-integrated PT has advanced to the point of initial clinical studies (32), with ongoing developments (**Figure 2H**) (33, 68–76). Several solutions are currently under pre-clinical exploration, including in-room/integrated or off-room/shuttle-based MR scanners. However, it is clear that further research and improvements are needed to define robust and reliable clinical MR-only workflows in PT. Depending on the clinical use case – such as initial treatment planning or dose re-calculation to assess adaptation needs – MR-based synthetic CT approaches do not necessarily need to match the accuracy of state-of-the-art fan-beam CT. A significant step forward would be the ability to trigger treatment adaptation through improved in-room imaging, allowing for re-planning and tailoring treatment in response to anatomical changes. At this stage, it remains uncertain whether MR-only PT treatment planning can achieve the same level of accuracy and precision as current CT-based methods, such as DECT-based direct SPR prediction. For the future potential of MR guidance in adaptive PT, further research is needed to assess its clinical applicability.

CT imaging with proton beams instead of X-rays is a promising technology to overcome the uncertainty in the conversion from X-ray attenuation to SPR (77–80). Despite ongoing research activities over the last decades, this technique is still in an early stage of development, i.e., first prototype proton CT scanners (**Figure 2I**) are constructed and experimentally validated, but a clinical system is currently not available (34, 35, 81). Current applications are mostly limited to head sizes, since today’s PT facilities equipped with cyclotrons are not capable to deliver proton beams with sufficient energy to cross body regions like abdomen and pelvis. Some dedicated synchrotron-based systems used for PT can overcome this limitation by reaching proton energies of 330 MeV corresponding to a range in water of 60 cm (82, 83). As demonstrated in a recent study (84), further improvements in image reconstruction are crucial to reduce the partly severe ring and streak artifacts in proton CT images. Furthermore, it was shown that DECT-based direct SPR prediction can achieve a comparable SPR accuracy as proton CT (84). Hence, it will not be trivial to demonstrate a benefit of proton CT compared to X-ray CT in terms of clinical outcome or cost efficiency.

Further non-volumetric imaging techniques may be combined with the above mentioned, in order to reconstruct the full range of intrafractional movements in the patient. Exemplary solutions are the visualization of external markers, the detection of internal markers or electronic transponders, online fluoroscopy for correlation of internal motion and external surrogate signal, ultrasound imaging and surface tracking (85). These motion management solutions are already quite established in image-guided routines and do not constitute per se an issue for their integration in a near real-time adaptive workflow, although stricter timing criteria and the accurate combination of multimodal information must be considered. A full review of motion management systems suitable in PT has been reported (16) and is beyond the scope of this review as these techniques do not provide 3D images suitable for dose calculation and (adaptive) treatment planning.

## 3 Online contour generation

One of the challenges associated with imaging for online plan adaptation is the rapid generation of contours on the 3D images, which provide the essential anatomy labels for the adapted plan and are crucial for performing dose-volume histogram (DVH)-based assessments. Despite being a long-known issue (86), this remains a major bottleneck for the development of an OAPT workflow (and online ART in general) (10) and is in general a time-consuming task also for the initial planning. Several strategies have been investigated to speed up the process and numerous commercial solutions for automatic segmentation of medical images and contouring (i.e., segmentation specifically aimed at generating contours for radiation therapy) are available (87–90). However, auto-contouring operations usually require manual checks, which often leads to further manual adjustments (91, 92). While this is acceptable for daily adaptive workflows, it places an unfeasible burden on the total adaptation time in NAPT. A fully integrated NAPT loop requires minimal user intervention, with ideally no involvement of a radiation oncologist in the treatment session. This is in contrast to what generally happens now for online photon-based ART, where the presence of the radiation oncologist is requested at least for some fractions (93). Moreover, not only the timing aspect, but also the accuracy of the auto-contouring process must be carefully addressed (94, 95).

Contour propagation based on (rigid or deformable) registration has already been investigated for clinical use in OAPT (12, 17, 96–98). This approach is well-established and already available in many commercial software products for treatment planning. However, it may be more suitable for tumor sites with minimal motion (e.g., brain, nasal cavities). Its application in more mobile anatomical areas or low-contrast regions, such as thoraco-abdominal tumors, could be more challenging. Additionally, many contouring tools incorporate prior knowledge, such as atlas-based or model-based segmentation. However, their performance depends heavily on factors like the contrast of the specific structure and the representativeness of the selected atlases or models (86), meaning these methods are not truly patient-specific. Several studies have explored the suitability of propagated contours in relation with intra-observer variability and need for manual adjustments with promising results (99–104), although patient-specific factors still play a significant role.

Auto-segmentation based on artificial intelligence (AI) is gaining increasing popularity and several approaches for auto-contouring are available or rapidly evolving. The development of deep learning (DL)-based segmentation in particular has driven a great number of studies lately (105). DL auto-contouring may in the future have the potential to outperform previous solutions, due to its capability to identify features in the data thanks to prior training and it may then be applicable to a broader range of tumor sites. However, current limitations stem primarily from the size and representativeness of the datasets used for training. Despite these limitations, many commercial software solutions offering DL-based auto-contouring are already available and are being extensively researched (106–108) and evaluated in clinical settings (109). Studies evaluating the impact of DL auto-contouring on contouring time have also been reported (110, 111), including comparisons with other auto-contouring techniques, such as atlas-based or DIR-based methods (112). Time savings compared to manual contouring have been shown, although no clear advantage for a specific method has been observed, and corrections are still required. Recently, strategies to account for the uncertainties related with the auto-contouring within the optimization process have also been proposed (113, 114). These methods could effectively mitigate the impact of contour uncertainties thus facilitating the adoption of automated contouring tools. However, further research is required at this stage.

Additional contouring best practices should also be considered. First and foremost, it is crucial to emphasize that contouring protocols must remain consistent between the planning CT and the additional images required for OAPT. Best practices include using pre-determined contouring strategies for specific structures, maintaining consistent naming conventions, and clearly differentiating between targets, OAR, and normal tissues. Further research is expected on the topic, with recent studies suggesting that manual corrections might be required only for the target contours and discarded for the OAR (115). A consensus has not been reached yet.

Moreover, the development of auto-contouring techniques for OAPT requires the definition of rigorous safety checks on the resulting anatomical structures. In fact, evaluating the acceptability of the contours and establishing suitable assessment metrics is another essential part of the process (95, 104, 116), and automatic safety protocols are of utmost importance for the application of auto-segmentation in OAPT. Final checks on the quality of the current structures can be considered as a part of the online quality assurance (QA) process and will be discussed in more details in the dedicated section.

## 4 Decision on online adaptation need

In conventional scheduled DAPT (**Figure 1A**), adaptation is performed at every treatment session. Alternatively, adaptation can be triggered (**Figure 1B, C**) while the patient is on the treatment couch, based on new 3D imaging, a re-calculated dose from the scheduled plan, or external signals such as treatment verification data. In this context, triggered NAPT (**Figure 1C**) can help reduce overall workload, since the adaptation would not be necessarily performed at every fraction. Online adaptation may be more efficient when performed only when clinically necessary or when a clear benefit is anticipated. This means, it is crucial to rapidly assess whether adaptation is deemed advantageous on the basis of the collected data (e.g., 3D image in treatment position, updated contours, re-calculated dose distribution and DVH parameters), before proceeding with the adaptive workflow. For photon-based ART, several workflows have been proposed depending on the timing of the adaptation, frequency of imaging and tumor characteristics (117). The approach of directly performing a plan adaptation, as exemplified in certain MR-LINAC workflows (24–26) and in conventional scheduled DAPT (17), poses a challenge as the online adaptation and related safety checks are time-consuming. Essentially, a timely decision-making with appropriate assessment tools that determine the adaptation demand has to be much faster than the plan adaptation and its QA itself.

A straightforward approach to assess the need for adaptation is the use of pre-determined thresholds and checklists that include anatomical and dosimetric information, such as variations in structure volume, center of mass, and DVH properties for the scheduled plan on the updated anatomy (12). In this context, dose calculation plays a key role, requiring a fast and efficient process.

Moreover, the development of automated decision support platforms for assessing the need for adaptation is expected to be greatly advanced thanks to AI. Several AI-based decision support systems have already been proposed in photon-based ART for different purposes, such as plan auditing (118), individualized treatment decisions for head and neck cancer (119, 120) and esophageal cancer (121), with the first commercial ART software platforms available for deciding when a plan adaptation is needed (122).

It’s important to note that the decision to adapt could be made at different levels depending on the availability of collected data, by extracting and processing relevant features. This could involve using only the anatomical information from 3D images, incorporating contour data, considering information from the dose distributions only, or a combination of these factors. Various AI techniques could be used to support this process. The capacity to extract pertinent information about the adaptation need from the images alone, without the necessity for initial plan recalculations or even updated contours, would facilitate a significantly more expedient decision-making process.

## 5 Online plan adaptation: optimization strategy and dose calculation

The online plan adaptation process involves two key aspects: the optimization algorithm used for the adapted plan generation and the dose engine used for the dose calculation. Relevant research in both areas is ongoing in the PT community. Several solutions to fasten the plan adaptation process have been proposed and comprehensively discussed (11). An overview of the most prominent OAPT plan adaptation algorithms and dose engines will be presented, with focus on near real-time implications.

In PT, the time required for standard plan optimization is largely determined by the number of robustness scenarios considered. In contrast, XRT can account for setup errors through an appropriate planning target volume (PTV) concept, and range uncertainties are negligible. Therefore, reducing optimization times is particularly critical for enabling fast online adaptations in PT compared with XRT. The main strategies for generating online adapted plans that align with the current anatomy can be broadly classified based on the underlying optimization technique and/or on the final goal of the adaptation (123). The principal plan adaptation strategies are: “standard” region-of-interest (ROI)-objective-based full re-optimization, constrained re-optimization retaining specific properties of the initial plan with or without the goal of reproducing the initial dose (dose restoration), and dose-mimicking-based full re-optimization (which can be tuned to mimic different dose distributions depending on the application). Furthermore, the use of plan libraries has been put forth as a potential strategy for OAPT (123). Additional adaptive strategies that could be applied after a partial delivery of the plan are particularly relevant for NAPT and will also be addressed down below. A detailed outline of the discussed strategies is presented in **Table 2**.

**Table 2:** Overview of the main proposed adaptive strategies for OAPT.

Adaptation strategy/technique	Implementation types	Suitable workflow application	Strengths (+)	Limitations (-)
Online ROI-objective-based “standard” full re-optimization *Optimize energies, spot positions and spot weights with the goal that the resulting dose distribution fulfils requested DVH criteria*	• Full new plan generation including beam setup according to the initial trial-and-error planning pipeline • Full re-optimization with pre-determined objectives for a daily template plan or, more specifically, with the initial plan optimization problem (12, 17, 96, 124)	Upfront online adaptation before starting daily delivery	+ Clinical standard for offline adaptation + More flexibility in the optimization + Effective against drastic anatomy changes + Pre-established optimization templates enhance comparability and simplify both initial and adaptive planning + Clinically proven: first reported DAPT treatments based on the full re-optimization of a daily template plan	− Longer optimization process − High level of online QA required
Online constrained re-optimization retaining specific properties of the initial plan *Adjust properties of given plan (e.g., modify spot position, energy and weight; add selected spots) to comply with new anatomy*	• Dose restoration, when the goal is to reproduce the initial dose distribution (125–127) • Range shift compensation, with or without spot weight tuning and spot addition (e.g., for a subset of spots), not necessarily replicating the initial plan dose (96, 128–131)	Upfront online adaptation before starting daily delivery	+ Related online QA expected to be less burdensome as specific plan properties (e.g., number of energy layers, dose distribution) are not/less modified + Keeping the initial dose distribution unchanged as in the dose restoration approach facilitates physician’s approval	− Unsuitable for drastic anatomy changes
Online dose-mimicking-based full re-optimization of a reference dose *Optimize energies, spot positions and spot weights with the goal to deliver a given 3D reference dose distribution to the current anatomy*	• Purpose defined by the reference dose to be mimicked ○ AI-predicted dose distribution (124, 132–134) • Rigid/Deformably propagated initial dose distribution (dose restoration approach) (124, 132, 135)	Upfront online adaptation before starting daily delivery	+ The opportunity to select different reference doses grants a flexible approach + Less burdensome than the traditional optimization workflow (based on ROI-objectives) + Mimicking of the initial dose as in the dose restoration approach may facilitate physician’s approval	− Depends on the ability of the mimicking algorithm to accurately reproduce a reference dose − AI-prediction of a reference dose is limited by the training dataset − Mimicking of the initial dose as in the dose restoration approach is unsuitable against drastic anatomy changes − High level of online QA required
Plan library *No plan optimization during the treatment session; just select the most suited plan*	• Pre-treatment creation of a variety of plans to counteract potential anatomy changes (136–139) • Creation of the plan library alongside the treatment course (140)	Selection of the daily plan before starting daily delivery	+ Potentially minor disruption to daily workflow as all plans are created beforehand and are not re-optimized online + Usual PSQA can be performed offline	− May not be representative of the current anatomy, ending up with further offline plan adjustments − Accurate plan-of-the-day selection criteria needed − Extra time and resources required for multiple plan creation and QA before the treatment
Online partial adaptation *Adapt only a not-yet delivered part of a plan but consider the sub-optimal dose delivered by the non-adapted plan portion*	• Dose mimicking of a reference dose considering the already delivered (background) dose (141, 142) • Range shift compensation considering delivered probing beam dose (143, 144)	Near real-time adjustments, e.g., triggered by treatment verification	+ Allows to address detected treatment deviations during delivery (verification-triggered adaptation) + Adaptation may be performed only when necessary (e.g., in presence of a detected deviation), thus reducing the related burden on the daily workflow + Time-saving against standard DAPT, allowing to start treatment with non-adapted field and adapt the remaining portion of the plan in the meantime	− Specific online QA checks required to ensure safety of the partial plan irradiation

The generation of a fully new plan (which entails new energies, spot positions and weights, and potentially also beam angles and/or air gaps) following the initial (ROI-objective-based) pipeline and the conventional trial-and-error approach is still the gold standard for offline adaptation (4, 15). However, it is a time-consuming process and may require several iterations for tuning the plan parameters by the user, before achieving an optimal quality. The goal of an online adaptation is not to achieve the best plan but rather a plan that reliably meets clinical constraints. Hence, methodologies to speed up full re-optimization are under research. For example, full re-optimization may be based on a pre-established template plan, without the need for online adjustment of optimization parameters, like objectives, beam angles, etc. To improve efficiency and ensure consistency, (non-)patient-specific ROI-objective-based templates can be applied to both initial and adapted plans, reducing manual input. Patient-specific templates can either apply the final objectives from the initial plan (124) or be created *de novo* during the pre-treatment planning phase (12, 17, 96). In the latter case, compared to standard clinical practice, the “daily” plan template allows for potentially more aggressive beam arrangements and robustness settings, though it increases pre-treatment workload.

Differently from full re-optimization, online constrained re-optimization techniques enforce direct adjustments of plan parameters (96, 123, 128–131), such as the spot energy/range, isocenter position and/or tuning spot weights for a subset of spots, rather than ROI objectives, offering a faster and less time-intensive online plan adaptation. In fact, in these adaptive methods, the spot positions and energy layers shall remain unchanged compared to the initial plans. This strategy is subject to some limitations, in that it may prove inadequate for compensating for significant anatomical variations and deformations. In such cases, the addition of energy layers or other more substantial modifications to the original plan may be required. These techniques have been applied with or without the goal of restoring the initial planned dose and have been previously referred to as simply “re-optimization” (11). In this context, “dose restoration” gained attention as an online constrained re-optimization technique specifically aimed at reproducing the initial dose distribution in adapted plans, for example in rigid dose restoration approaches, such as the one described by Jagt et al. (125) for prostate cancer. Since then, it has been investigated for other tumor sites (124, 126, 127, 132, 135) in more broadly defined approaches aimed at reproducing the initial dose distribution or the initial plan quality in adapted plans. The key motivation for dose restoration is its potential to save time by reducing the impact of contouring, as the initial structures could be used on the current anatomy. Sometimes, it is claimed that constrained online re-optimization techniques simplify online QA by preserving certain properties of the initial plan, but this cannot be the main argument, as a high level of QA is still required to ensure a safe adapted treatment. Rigid dose restoration is achieved by propagating the initial structures and restoring the initial dose distribution against density changes, assuming no differences in position or shape of the targets and OAR. This replicates the “static dose cloud approximation” from conventional radiotherapy, where patient shifts and density variations have minimal impact on isodose shape and position. However, applying this concept to PT has limitations. Despite efforts to enhance robustness (126), the assumption that the initial dose distribution suits the current anatomy fails in many cases. Complex anatomical changes, such as deformations, position variations, or poor registrations, can undermine the technique’s effectiveness (127). It is thus not ideal for abdominal or thoracic sites, which may require NAPT interventions. Additionally, current time performances do not meet daily OAPT or real-time requirements, suggesting a need for faster hardware or alternative solutions for clinical viability (127).

Dose mimicking has been proposed (124, 132–134) as an alternative adaptive plan optimization strategy, where the optimizer aims at generating a completely new treatment plan (energies, spot positions and spot weights) that delivers a certain given reference dose distribution, similar as to what has been initially investigated in the context of XRT (145). The dose mimicking operation can have different goals, depending on the reference dose to be replicated. For example, the reference dose may be an AI-predicted dose on the new anatomy, in a “dose prediction plus mimicking” approach (132, 133, 146, 147). However, the reference dose may also be the dose distribution of the initial plan copied to the new image (132). This last approach aims at restoring the initial dose cloud and can therefore be regarded as another technical realization of dose restoration. Deformed dose restoration based on dose mimicking has been investigated as a potential solution to address some of the limitations of the rigid approach. The aim is to restore a dose cloud that has been adapted to the new anatomy through the use of guided dose deformation (135), where specific control structures serve as guiding reference for the deformation.

An explored alternative to online plan adaptation is the use of pre-determined plan libraries, where multiple treatment plans are created in advance to account for potential anatomical variations (e.g., organ filling changes). These plans undergo the elaborate routine QA before they can be selected for delivery based on the patient’s anatomy at each fraction, minimizing workflow disruption. This approach, which hence does not represent a truly online plan adaptation, is not expected to become standard clinical practice in PT (148, 149). Several retrospective planning studies across different tumor sites exist (136–139), and a first clinical application for an intra-thoracic tumor has been reported (140). In silico studies have explored various methods for generating plan libraries, such as motion modeling, marker tracking, and adjusting setup margins using retrospective control CTs. In the reported clinical case, the plan library was built progressively during treatment based on acquired control CTs. Inherent limitations of the strategy prevent a broader adoption of plan libraries. The plans available in the library may not include the full range of geometry variations and may end up being not suitable for the daily delivery; this is particularly relevant for NAPT aiming to tackle unexpected intrafractional changes.

Adaptive strategies as the ones described so far focus on the adaptation of the entire plan before starting any beam delivery in the daily treatment session. To streamline daily adaptive workflows, more flexible approaches are desirable – such as selectively adapting only portions of the plan, while a part of the fraction dose is already delivered. This can enable parallel operations, thereby improving overall efficiency (141). Dealing with a sudden change detected during delivery as in the foreseen near real-time adaptive setting is an additional challenge. The employment of online *in vivo* treatment verification will not only offer a tool to control the compliance of the delivered (adapted) treatment, but will also enable a transformation in the way the online adaptation is applied. In fact, the detection of a treatment deviation above an established threshold may serve as a signal to trigger a subsequent image acquisition in treatment position and, ultimately, an adaptation of the plan in the fully closed near real-time adaptive loop. This so-called adaptation triggered by treatment verification might in principle happen at any time during the delivery, propelling the need for plan adaptation strategies to be employed mid-delivery or even multiple times during delivery. In this context, the concept of a “partial adaptation”, whereby only a portion of the plan is adapted, is also of paramount importance. Without the ability to adapt only the undelivered portion of the scheduled plan in response to a detected treatment deviation and to consider the delivered dose up to the trigger, NAPT triggered by verification would be unfeasible. Planning strategies that introduce a partial adaptation for PT have recently been proposed (141, 143) and further investigated in combination with the potential trigger information from a treatment verification system (142, 144).

A fast dose calculation is essential within the iterative adaptation algorithms and beyond. Dose calculation plays also a key role in multiple steps of the online adaptive workflow, including initial plan recalculation on the current anatomy for adaptation decisions and independent dose verification for QA (11). Unlike photon dose calculation, proton dose calculation presents unique challenges. Early clinical approaches in PT relied on analytical algorithms such as pencil beam and ray casting, which provided fast and efficient performance (150–152). These methods have been explored for the use in OAPT, with strategies proposed to improve their accuracy (153–155). However, their limitations have been well-documented, particularly when compared to Monte Carlo (MC)-based dose calculation, which offers greater precision (150, 156). MC-based dose calculation represents nowadays the gold standard regarding accuracy, is broadly clinically applied and MC-based dose engines have been incorporated into all relevant commercial TPS (150, 151). Significant efforts have been made to develop powerful hardware (157, 158) capable of faster performance, in combination with novel MC-simulation strategies (157, 159). While MC-based dose calculation still poses challenges for near real-time adaptation due to its time requirements, it is increasingly being used in OAPT workflows. This is evident in its application for independent pre-delivery checks of online adapted plans and log-file-based post-delivery QA, as will be discussed in the next section. Additionally, its superior accuracy and anticipated improvements in time performance make it the preferred method for proton dose calculations.

Finally, several studies propose novel AI-based approaches for proton dose computation, such as knowledge-based planning (160), long short-term memory networks (161) or transformers (162). Many of these approaches aim at achieving the same level of MC accuracy at shorter calculation time (157, 163–165). There are still limitations in applicability to different sites or different field arrangements than the ones included for the training of the AI algorithms. Hence recent works focus on generalizable algorithms that could return MC accuracy in sub-second speed, which could be potentially relevant for near real-time applications (166), if not during optimization then at least for fast secondary dose calculation in the online QA (see next section), including AI-based dose calculation models specifically designed for OAPT (167–170).

## 6 Pre-delivery QA of the online adapted plan

QA of the treatment plan, so-called patient-specific QA (PSQA), is an essential step to ensure the treatment safety. PSQA aims at checking 1) that the calculated dose distribution for the plan is correct, 2) the correct data transfer between the TPS and the delivery system, and 3) the deliverability of the plan (171). In most PT centers, PSQA still involves dosimetric measurements for plan deliveries to water and water-equivalent phantoms, which are time-consuming and limit the availability of the treatment room. Recently, the combination of log-file-based verification with an independent dose calculation became a valid alternative for the above-described three aims, that already found its way in clinical application in several centers (14, 17, 172). It is clear that, within the context of OAPT, phantom-based PSQA procedures are unfeasible due to the strict time constraints (10, 11), requiring new strategies for measurement-less PSQA that can be performed during the treatment session and before delivery. Therefore, the trend towards phantomless PSQA is synergistic with the implementation of OAPT. Still, an offline (phantomless) PSQA and a PSQA of a treatment plan that was just generated a few seconds or minutes before application represent different conditions and requirements. A dummy-irradiation to generate a log file is not possible for OAPT and therefore the log file results will only be available after patient treatment. Therefore, QA checks that can be performed before delivery are crucial. One key component is a secondary dose calculation using an independent dose engine. Another essential element is the implementation of ‘sanity checks’, which are conducted at various stages of the workflow before the online adapted plan is approved and delivered. They confirm the plausibility of adapted plan changes and verify data integrity before treatment. Unlike conventional pre-treatment PSQA, online adaptive workflows have access to additional information, particularly the initial plan as reference. This enables two types of verification: 1) Hard checks, which correspond to standard pre-treatment QA procedures and require an exact match with the reference plan (such as patient name, etc. 2) Soft checks, which assess deviations from the reference plan within defined tolerances. For soft checks, defining appropriate thresholds is complex and requires careful consideration to ensure clinical safety and treatment accuracy.

For example, soft checks can be applied to evaluate the obtained structures on the actual anatomy, ensuring that the online contouring process has been performed correctly. This is particularly important when contouring is automated using dedicated software, which is desirable given the time constraints of OAPT. Exemplary structure parameters can be selected to be part of this structure pre-delivery QA to assess intensity and geometry, like the center of mass, the volume, the average CT numbers of specific structures, etc. (12, 173). For the online adapted plan, different inputs can be used and assessed in secondary dose calculations, depending on the evaluation’s specific goal. Machine steering files have been proposed as inputs to evaluate the correct data transfer and deliverability, as they contain treatment plan information converted into delivery system instructions (174). However, access to these files may not always be feasible. In such cases, an alternative approach is to run the evaluation in the TPS, use external software, or work with exported DICOM files. Selected plan parameters, such as energy layers, spot numbers, cumulative monitor units, etc. can then be evaluated with hard or soft checks, depending on the accepted tolerance relative to the initial reference plan, for which standard PSQA was performed. Automation, safety and speed are central to the online PSQA process, requiring seamless integration of multiple QA levels and parallel execution of tasks. For example, the online PSQA in standard DAPT can be performed alongside patient setup and setup verification. The aforementioned QA procedures and introduced sanity checks do not require actual plan delivery and are well-suited for an online (near real-time) timeframe.

Proton range assessment using range probing is an additional pre-delivery QA method, also applicable to OAPT workflows, and is currently under translational research. Its purpose is to verify the accuracy of the SPR prediction used in treatment planning (175, 176). Range probing utilizes high-energy, low-dose proton beams (177) to measure the remaining energy of protons after traversing the patient. This is achieved using either multi-layer ionizazion chambers (MLIC) (178) or flat panel detectors (179), allowing for a direct comparison between the measured energy loss and the simulated values from the planning CT or a pre-treatment image of the current fraction, when available. In the context of OAPT, range probing is primarily intended to validate CBCT-based SPR prediction (179–181). Following initial proof-of-principle and phantom-based validations, the first proof-of-concept clinical implementation has been performed (19). For widespread clinical adoption, integration into PT systems would be necessary, potentially achievable through flat panel detectors. However, no medical product is currently available for routine clinical use.

## 7 Online treatment verification and detection of deviations during delivery

*In vivo* treatment verification during delivery has specifically two main goals: 1) to provide a safety net, checking that the adaptation worked out as intended; 2) to detect deviations due to inter-/intrafractional changes in the anatomy that may trigger NAPT. It is also highly desirable when aiming at reduced target margins. The first-to-clinic daily adaptive workflows do not include treatment verification, and are based solely on the detection of anatomical changes on the daily image taken before the delivery is started (12, 17, 18). As previously introduced, the detection of a relevant treatment deviation during delivery and the ability to act on it promptly is essential for near real-time adaptation, making online treatment verification a crucial step in the workflow – effectively closing the feedback loop. A number of techniques have been proposed for the verification of range *in vivo* (**Table 3**). The investigated *in vivo* range verification techniques have been differentiated depending on the measured information (e.g. positrons or prompt gamma rays), timing (online or offline), dimension (1D, 2D, 3D) (1) and applicability (general or specific to a tumor location) (177). The most relevant ones are discussed below in relation with potential applications within a near real-time adaptive workflow.

**Table 3:** Overview of the close-to-clinic in vivo treatment verification approaches for PT in terms of their suitability for near real-time adaptation.

Verification technique	Flavors	Timeframe	Integration in the workflow	Clinical readiness	Strengths (+)	Limitations (-)
PET-based (PT-PET)	• In-beam PT-PET • In-room PT-PET • Offline PT-PET	A few minutes dependent on the activity build-up of positron-emitting isotopes	In-beam PET with smallest effect on clinical workflow (additional time need for acquisition after delivery), while in-room PET reduces patient throughput and offline PET requires additional resources (e.g., close-by PET scanner)	In clinical studies for treatment verification of proton and carbon ion treatments with in-beam detectors relying on common positron emitters. No medical product available	+ Broad clinical experience (with hundreds of clinical applications) + Information available shortly after delivery + Commercially available detector technology from diagnostic PET can be utilized	− Information from different spots and fields not differentiable due to stochastic decay mechanism − No real-time capability − Bulky and complex instrumentation − Biological wash-out − Limited accuracy (also due to time until positron decay and low counting statistics)
Prompt gamma-based (PGTV)	• PGI • PGT • PGS • Multi-feature	Online; prompt gamma rays produced almost instantly (few ns)	Easily integrable with minimal effect on clinical workflow (acquisition during delivery)	In clinical studies for online verification (PGI and PGS). No medical product available	+ Available during delivery + Produced quasi-instantaneous allowing spot-wise resolution and evaluation + Broad clinical experience (with hundreds of clinical applications) + Different evaluation approaches available (binary classification for relevant treatment deviation or eventually even 3D dose reconstruction)	− Complex and partly bulky instrumentation
Range probing	• MLIC-based • Flat-panel based	Before treatment delivery	Additional time needed to deliver and evaluated the range probing spots	In clinical study for online verification in at least one center. No medical product available	+ No issues with multi Coulomb scattering (measurement compared with expectation) + Information available before treatment (e.g. on CBCT validation) + No advanced detector technology required	− Additional time required in treatment room. − No differentiation, if detected changes affect treatment field or not − Not during dose delivery (changes after range probing stay undetected)

For completeness, range probing is included given its advanced status, even though it is generally applied before delivery of the therapeutic treatment and not during delivery, differently from PT-PET and PGTV.

One of the first *in-vivo* verification techniques studied was PET imaging during particle therapy delivery (previously also referred to as PT-PET) (182–184). Positron emitters (^10^C, ^11^C, ^15^O among the most commonly used) are produced via nuclear interactions between the incident protons and atoms of the patient’s tissue and PET-based treatment verification relies on the coincident detection of 511-keV photons produced by the annihilation of the emitted positrons with electrons. Unlike particle therapy with heavier ions, when using proton beams positron emitters can only originate from the target atoms, rather than from projectile fragmentation. Because the nuclear interactions and thereby the positron emission are closely related to the dose deposition, the positron activity can be used to approximate the proton beam range, usually by comparing the measured activity distribution with an expected one derived from the treatment plan (185) and deriving a range deviation. Given that PET imaging is a well-established diagnostic technique, there has been considerable interest in adapting it for the use in *in vivo* treatment verification (186). Several solutions have been proposed, each with their specific advantages and disadvantages: online in-beam PT-PET involves PET imaging directly after each field delivery, and sometimes during irradiation; online in-room PT-PET entails PET imaging with an in-room PET scanner after the patient is moved to the scanner position post-treatment; offline PT-PET requires transferring the patient to a nearby room, where PET imaging is performed using a standard diagnostic PET scanner (187). While offline PT-PET can rely on standard diagnostic systems, online PT-PET systems are usually dedicated in-house developed systems that can be used in the room while the patient is on the couch, hence they must respect certain geometrical constraints (188).

While many research groups have investigated PET-based range verification in clinical studies (184, 188, 189), and several hundred patients have been monitored at a few pioneering institutions, the method has not found its way into broad clinical application. This is probably due to the inherent limitations of the approach: due to the half-life time of the positron emitters in the range of minutes for the most prominent positron emitters (20 min for ^11^C, 2 min for ^15^O and 19 s for ^10^C) a spot-wise evaluation is not possible, as the signals from different pencil beam spots and even from subsequent treatment fields overlap each other. Additionally, the biological wash-out effect (190), which describes the movement of positron emitters between their generation and decay, leads to a blurring of the original activity distribution. Intrafractional movement needs to be monitored and considered correctly to correlate the locations of positron emitter generation and dose deposition with the location of positron emission (191–193). The positron activity produced is relatively low, around 6.6 kBq per Gy and cm³ (194), compared to 10–100 kBq/cm³ in diagnostic nuclear medicine. As a result, the derived signal is heavily influenced by statistical uncertainty, and interpreting clinically relevant changes at the dose distribution level remains challenging, particularly due to the differences in activity and dose distribution, especially when using proton beams.

Despite its limitations and restricted applicability, research in the field of PT-PET continues. An experimental intrafractional adaptive PT workflow based on PET verification has recently been proposed and investigated pre-clinically (144). Additionally, the use of short-lived isotopes, like ^20^N with a half-live of 11 ms (195, 196), has been suggested, however their activity concentration is about two orders of magnitude below that of conventional emitters mentioned above. Further developments of in-beam PET detectors (197), application of AI to the obtained PET images (198), time-of-flight PET (199, 200) and combined techniques (201) have also been reported.

Currently, the most promising *in vivo* treatment verification technique is the detection of prompt gamma (PG) rays, i.e., photons that are almost instantaneously (<ns) produced after nuclear interactions of the beam with the nuclei of the traversed tissues (1, 177, 202–204). These emitted PG rays are correlated with the depth-dose profile of protons. In general, the timeframe of PG ray’s emission makes PG ray-based treatment verification (PGTV) particularly well-suited for NAPT. Differently from PET-based treatment verification, PGTV is not affected by activity build-up or decay times, hence it could provide an instantaneous information about treatment deviations, and the PG rays have a low probability to undergo secondary processes before reaching the detectors. Moreover, PGTV may be employed to continuously monitor the delivery.

The use of PG rays for proton range verification was first proposed in 2003 (202). A range of different PGTV techniques is now actively studied, the main ones comprising PG imaging (PGI) (205), PG timing (PGT) (206) and PG spectroscopy (PGS) (207). PGI aims at recording the spatial distribution of the emitted PG rays, PGT is based on their temporal distribution, and PGS relies on the local energy spectrum of the emitted PG rays, relating the PG spectrum to the proton-energy-dependent cross section of the respective nuclear reactions. With improvements in detectors, different domains of information could also be combined, enabling so-called multi-feature treatment verification.

PGI is currently the most advanced of the available PGTV techniques. It is in clinical application since 2015 (208, 209) and has since been applied in more than 470 field deliveries within a retrospective clinical study (210), proving the feasibility under realistic conditions. PGS is in clinical application since 2020 (211). Recently, a multi-institutional comparison of PGI and PGS has been performed under controlled conditions, providing standard conditions and methodology that allow fair comparisons and performance characterization of different PGTV systems, which are hopefully used in future studies proposing novel PGTV techniques (212). Accuracy of PGTV systems on spot level is currently around 2–3 mm for range verification, given a high spot dose (205). While all approaches measure primarily the PG information per pencil beam spot, there are quite different approaches of evaluating this raw data to conclude on clinically relevant treatment deviations. Usually, first a spot-wise range shift is determined by comparing the measured PG signal with an expected PG signal (213, 214). However, in real-world application that spot-wise information has limited accuracy, especially for low weighted spots, but more importantly a conclusion on clinically relevant treatment deviation is difficult from range information at the spot-level. One proposal is to reconstruct the 3D dose distribution from PG information, while another approach is to condense the PG data into a classification that identifies relevant treatment changes (either binary, such as yes/no, or multi-class, distinguishing the source of error). The classification method has been successfully tested and validated on clinically acquired PGI data for prostate treatments (215) achieving very promising results. Furthermore, the classification approach has been explored on different complexity levels for head and neck treatments (216, 217). Yet another approach, called spot boosting, proposes to manipulate the clinical optimized treatment plan by boosting the dose for a limited number of pencil beam spots and to irradiate those spots first, to selectively use them for PG range verification at those selected spot locations (218).

**Figure 3:**
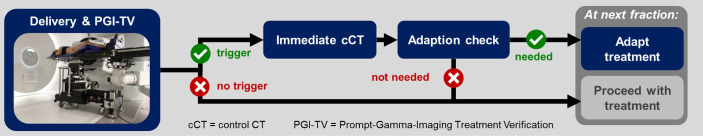
Schematic representation of the envisioned workflow for the DEPICT clinical trial. Treatment delivery is regularly monitored using PGI. If relevant treatment deviations are detected, a cCT scan acquisition is triggered to assess the need for plan adaptation immediately after the fraction dose delivery. If adaptation is required, an updated treatment plan is created offline for the next fraction. Otherwise, treatment proceeds with the next fraction as usual. (Courtesy of Jonathan Berthold).

Despite its proven clinical applicability, no medical products for PGTV are currently available. A significant technological challenge is the integration of the PGTV system near the isocenter (e.g., direct attachment to the nozzle or gantry). However, with several clinical studies (219) underway, focusing not just on technical proof-of-concept but also on clinical impact and treatment intervention – particularly in the context of OAPT – PGTV is well-positioned to play a key role in future OAPT-ready treatment systems. For example, an upcoming clinical interventional trial (DEPICT) planned at OncoRay, Dresden, aims to evaluate the capability of PGI to trigger an online intervention. In this case, the acquisition of a CT scan will be triggered if PGI detects a clinically relevant treatment deviation, based on a model (215) trained on paired PGI and ground-truth dose distribution data from a retrospective patient cohort (**Figure 3**). While this study may not involve online adaptation, it represents an important first step toward establishing a closed feedback loop reliant on treatment verification (in this case, PGI). This progress is also driving the investigation of novel PGTV techniques (220).

Recently, treatment verification based on ionoacoustic imaging, which utilizes the generation of ultrasound waves from the periodic temperature increase in tissue caused by the pulsed proton beam, has been studied in simplified, non-clinical experimental settings (221–228). The localized energy deposition from a proton pulse around the Bragg peak causes a local increase in temperature and pressure. This generates an acoustic wave that propagates isotropically and that can be detected when it reaches the patient’s skin. By analyzing the time of flight of the ionoacoustic signal, the position of the Bragg peak can be determined. However, treatment verification using ultrasound detection is still in its early stages and faces several challenges, including the propagation of ultrasound through heterogeneous tissues with varying sound speeds, reflection and attenuation, transducer characteristics, and background noise. These factors significantly degrade the signal-to-noise ratio under realistic conditions, raising questions about its clinical applicability.

In addition to using dedicated systems during the delivery of treatment to monitor its compliance, log-file-based dose calculation offers the opportunity to check the actual delivered dose after the delivery is completed (229, 230), or even mid-treatment in recently reported investigations (231). Since log files are mostly not employed at the time of treatment and usual applications aim at reliable post-delivery quality controls and dose accumulation, they will be discussed in the next section dedicated to tasks “beyond” the closed OAPT feedback loop.

## 8 Beyond the loop

The advent of NAPT offers the potential to finally close the long-awaited feedback loop during the treatment session (3). This entails a number of essential tasks and requirements that extend beyond the daily treatment workflow and that need to be carefully addressed.

First, the technological realization of a NAPT solution with a closed feedback loop requires changes in soft- and hardware. While introducing new medical products is a standard procedure within the medical technology industry, this process is not typically applied to in-house developments in translational research. In these cases, institutions face significant challenges to be compliant with the Medical Device Regulation (MDR) in Europe or the regulations by the Food and Drug Administration (FDA) in the United States. Especially the MDR requirements not only demand substantial resources, but they can also delay the clinical application of new technologies. At this stage, it is highly advisable for pioneering institutions to seek structured guidance from MDR experts as early as possible to streamline the development process and ensure compliance. Additionally, adopting a pragmatic approach is essential in determining the depth of legal requirements to be met (e.g., risk analysis, documentation). Medical physicists are well-positioned to make these pragmatic decisions, as they have long been responsible for risk assessment and patient safety, well before the introduction of the MDR. For in-house developments, the goal should be to integrate this expertise and methodology into the MDR process as seamlessly as possible, while minimizing the burden of additional bureaucracy. In this context, it is important to recognize the priorities of the European Society for Radiotherapy and Oncology (ESTRO) within the framework of the European Alliance for Medical Radiation Protection Research (EURAMED) (232), which emphasize interdisciplinary collaboration to improve clinical outcomes and minimize side effects. ESTRO advocates for the clinical implementation of new radiotherapy technologies, as a means of advancing precision medicine. This focus aligns with ongoing research and regulatory efforts aimed at supporting clinical trials and ensuring that emerging technologies like OAPT are thoroughly evaluated for their clinical efficacy, time efficiency, and cost-effectiveness.

Secondly, as anticipated, the introduction of log-file-based dose recalculation allows to perform additional checks on the treatment compliance. They usually lie outside of the “online” loop, since they are mostly performed only after the clinical delivery is concluded. This “log-file-based QA” relies on treatment delivery log files from machine’s internal beam monitors to reconstruct the delivered dose under actual treatment conditions (gantry angle and patient-specific anatomy). This approach eliminates the necessity for phantom measurements at a fixed gantry angle of 0° and as such has already found its successful way in routine clinical application with available medical products (233). It has been investigated in numerous workflow scenarios in PT, including fully automated PSQA solutions (14), and pioneering OAPT workflows (12, 17). Log-file-based 4D dose reconstruction for the irradiation of moving targets has also been investigated (234), and novel methods that can be applied within the delivery (231) offer a powerful tool for monitoring the delivered dose during treatment. The real-time availability of the delivered dose would allow to monitor the impact of movement and verify the efficacy of employed motion mitigation techniques. Moreover, it is an important step towards the introduction of near real-time adaptations relying on information on the actual delivered dose. In addition, the availability of log files in OAPT can help improve the dose accumulation quality (10, 11). Although this would be a powerful resource to evaluate treatment outcome and make predictions regarding adaptation needs, dose accumulation is currently not used routinely in clinical practice, due to the related uncertainties in image and dose registration having a great impact on the final dose distribution (10, 11, 235, 236). Despite its scarce clinical application (148), the interest towards dose accumulation in research is increasing, in relation with the development of OAPT. NAPT may benefit greatly from the availability of such a tool. Many recent studies have explored strategies to account for registration uncertainties in dose accumulation (237–240), even including DL, which may be a great asset for future applications of the accumulated dose (241).

Lastly, an efficient interconnection between the involved systems in the workflow is crucial for the implementation of the proposed near real-time adaptive closed feedback loop. Research to date has focused on the single tasks (10, 11) and the clinical landscape is actually quite fragmented (149), with only very first clinical DAPT applications reported in non-complex settings (17). The establishment of OAPT workflows is an open problem demanding multidisciplinary and multivendor collaborations. The process requires the overall development of novel components of hard- and software or the integration of novel modules within established systems (e.g., oncology information system (OIS), TPS). These systems must perform key functions for online adaptive workflows, such as real-time imaging integration, automatic adaptation of treatment plans based on changes in the patient or tumor, dynamic re-contouring of targets and OAR, online QA operations, incorporation of data from real-time treatment verification, etc. Additionally, they must seamlessly combine data from various sources (e.g., imaging, treatment logs, and contouring software) and ensure that different platforms work together effectively to enable timely, safe, and efficient treatment delivery. Recently, a few academic-industrial consortia have emerged to work on potential solutions addressing the different tasks in OAPT workflows, highlighting the great interest in the topic and the push towards its clinical implementation (242, 243). Among these, the ProtOnART consortium (243) stands out for the direct collaboration between academic institutions (OncoRay, Dresden and PARTICLE, Leuven) and industries (IBA, Louvain-la-Neuve and RaySearch AB, Stockholm), with the goal of providing an OAPT solution that could be accessible to multiple centers, rather than being limited to a specific in-house application. Unlike other initiatives focused primarily on research, the ProtOnART consortium has a strong translational foundation, with the goal of treating its first patients in 2026, focusing on lung and esophagus cancer in the first step. Specifically, the consortium’s short-term objective is to establish an efficient online daily adaptive workflow for PT demonstrated in clinical practice by its clinical partners. The ultimate goal of ProtOnART, however, is to enable NAPT, where plan adaptations are made during or between proton field deliveries, bringing this technology into clinical reality and thus making it highly relevant for the purpose of this review.

It is crucial to emphasize that the implementation of efficient data streams and the timely performance of tasks will be essential to achieve the overall objective of NAPT. For example, the possibility of performing parallel tasks is essential, such as initiating the assessment of adaptation needs while the contouring process is still ongoing, commencing the online plan adaptation process before the final contours have been approved, or sending the online adapted plan to the delivery machine before the final results of the online QA process have been obtained. Together with this, a thorough documentation of the online adaptive session is demanded which goes beyond the current standards for reporting and documenting PT treatments (244), with new guidelines currently under development. This documentation might take the form of extended plan reports, dedicated to specific tasks. Image-related documentation could include imaging protocols, the employed image registration (if images are not taken at the isocenter), contouring protocols documenting how different regions of interest were obtained and if manual adjustments were necessary. The reason for the online plan adaptation should also be reported, for example, by noting the DVH criteria that have triggered an automated adaptation due to exceeding predefined thresholds or other clinical motivations. The initial plan used for dose recalculation and assessment, details of the online plan adaptation (e.g., robustness settings, optimization settings) and the results of the DVH-metrics comparison could all be part of the online adapted plan documentation. Moreover, reports of the online QA containing the results of the performed sanity checks should be automatically provided. Finally, the timings for each step in the workflow, which would provide a basis for performance evaluations, should also be recorded. Whenever applicable, documentation should be appropriately stored in the OIS.

It is also important to recognize that while triggered NAPT has the potential to streamline certain aspects of the workflow, it may simultaneously introduce new complexities both inside and outside the treatment room. These include increased demands on staff for monitoring, decision-making, and QA, as well as the need for additional training to effectively operate new clinical tools and automation systems. All professional groups involved in the workflow would be affected, similar to current practices in online adaptive XRT: physicians would face additional workload for reviewing online-generated contours; medical physicists would take on responsibilities for online QA verification and offline review of the adapted plan; and radiotherapy therapists (RTTs) would need to manage a modified in-room workflow, potentially including increased responsibilities like in RTT-only workflows (245, 246). In this context, it is essential to establish clear intervention protocols that define decision rules, intervention thresholds, and corresponding actions. Such structured approaches enable efficient RTT-led workflows, ensuring that physicians and medical physicists are only required to intervene when unexpected or out-of-tolerance situations arise. Despite these initial challenges and the associated learning curve, it is expected that with growing clinical experience and ongoing technological advancements, the workflow will become more streamlined, thereby reducing the additional training and workload over time. In this regard, valuable insights can be drawn from the experience gained in implementing online adaptive XRT (247).

## 9 Summary and outlook

**Figure 4:**
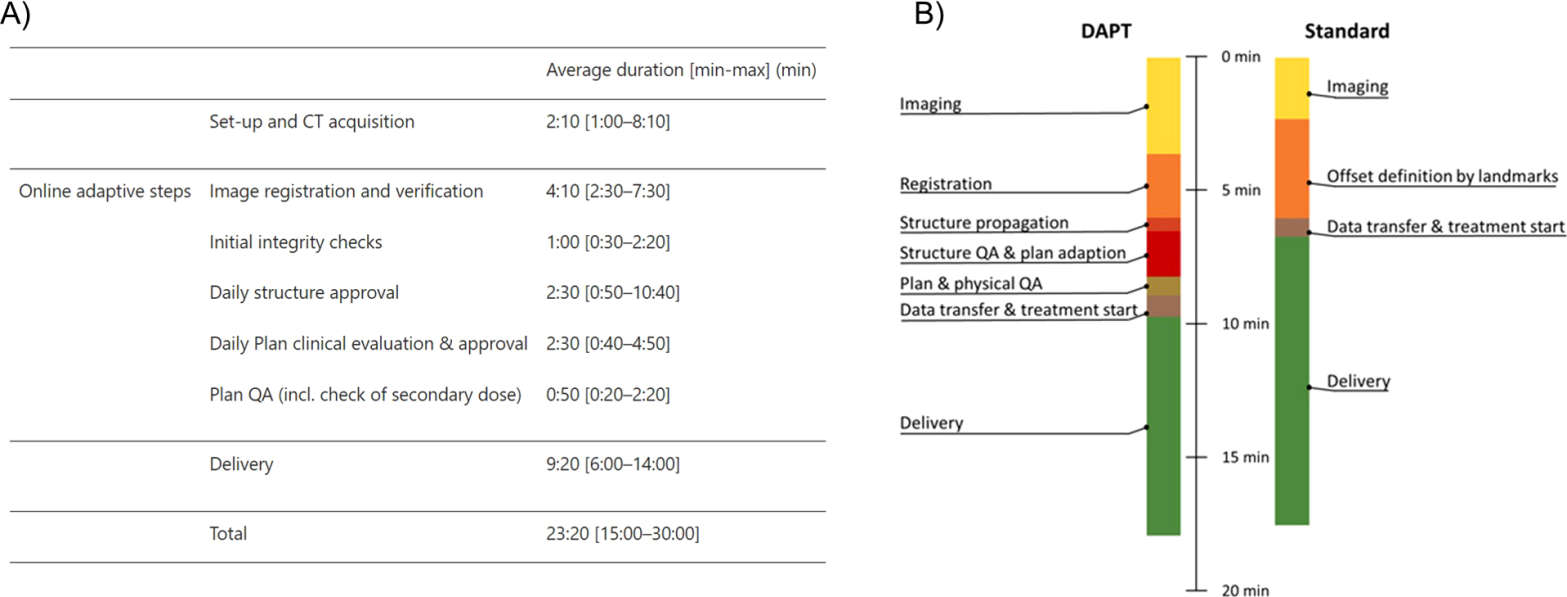
Timing reports for the first clinical applications of DAPT **(A)** and previous experimental tests of the same workflow **(B)**, reproduced from (17) and (12) under a CC-BY 4.0 license, respectively. Panel **(A)** details the timings for the first reported clinical fractions, while panel **(B)** shows the average timings from the experimentally delivered fractions. The first clinical results align closely with the experimental tests, demonstrating the feasibility of the workflow within a typical daily appointment timeframe.

OAPT is on the path to becoming a clinical reality, but it still remains greatly limited at present. The driving force behind this progress lies in unmet clinical needs, such as the growing demand for more precise and timely treatments and the inclusion of previously undertreated entities. These needs are prompting ongoing research and technological advancements aimed at enabling faster and more efficient adaptations. This review provided a comprehensive overview of the key components and tasks essential for the successful implementation of an OAPT workflow, ranging from daily adaptive to near real-time, with main focus on the latter. First in-house clinical implementations of daily adaptive workflows have already been successfully carried out (17), indicating that the necessary tools and frameworks for standard DAPT workflows are already in place. The first clinical application of DAPT has confirmed the feasibility of performing OAPT within a reasonable time frame during a treatment session (**Figure 4**). Moreover, interest is increasing in developing solutions that extend beyond in-house applications (243). Additionally, the establishment of a closed feedback loop for OAPT in clinical settings is already within reach, thanks to advancements in treatment verification techniques, as outlined. These developments create a solid foundation for accelerating treatment timelines, eventually moving towards near real-time applications.

At the same time, the requisite effort demands for the definition of clear guidelines and clinical approaches to be carefully redesigned. For a widespread use, several aspects of OAPT must be carefully assessed. This involves a multidimensional evaluation, considering factors such as clinical benefits, time efficiency, cost-effectiveness, and more.

The lack of evidence and the scarcity of clinical outcome data on PT represent in general a significant challenge to the establishment of solid indications for this modality, that needs to be overcome. The issue is thoroughly discussed in the PT community (248–251) and recently results of several completed clinical studies (including randomized clinical trials) have emerged with positive results showing improved clinical outcomes, reduction in toxicity, mitigation of acute and late effects, and consequential reduction in total cost burden (252–254). In this context, it is important that OAPT be introduced within clinical studies from the outset to assess its benefits across all dimensions. This includes evaluating its ability to enhance clinical outcomes, its competitiveness with XRT, its potential to enable hypofractionation, and its role in treating new tumor entities.

Economic factors are another critical consideration in the development of efficient OAPT and represent a significant limitation within the broader context of PT, especially when compared to XRT (250). While evaluations of opportunity costs for online adaptive treatments have already been presented for XRT (255), similar analyses are still lacking for PT. However, as the availability of PT centers continues to increase (252) and the number of patients grows, more clinical data will emerge, creating valuable opportunities for the development and effective use of OAPT.

**Figure 5:**
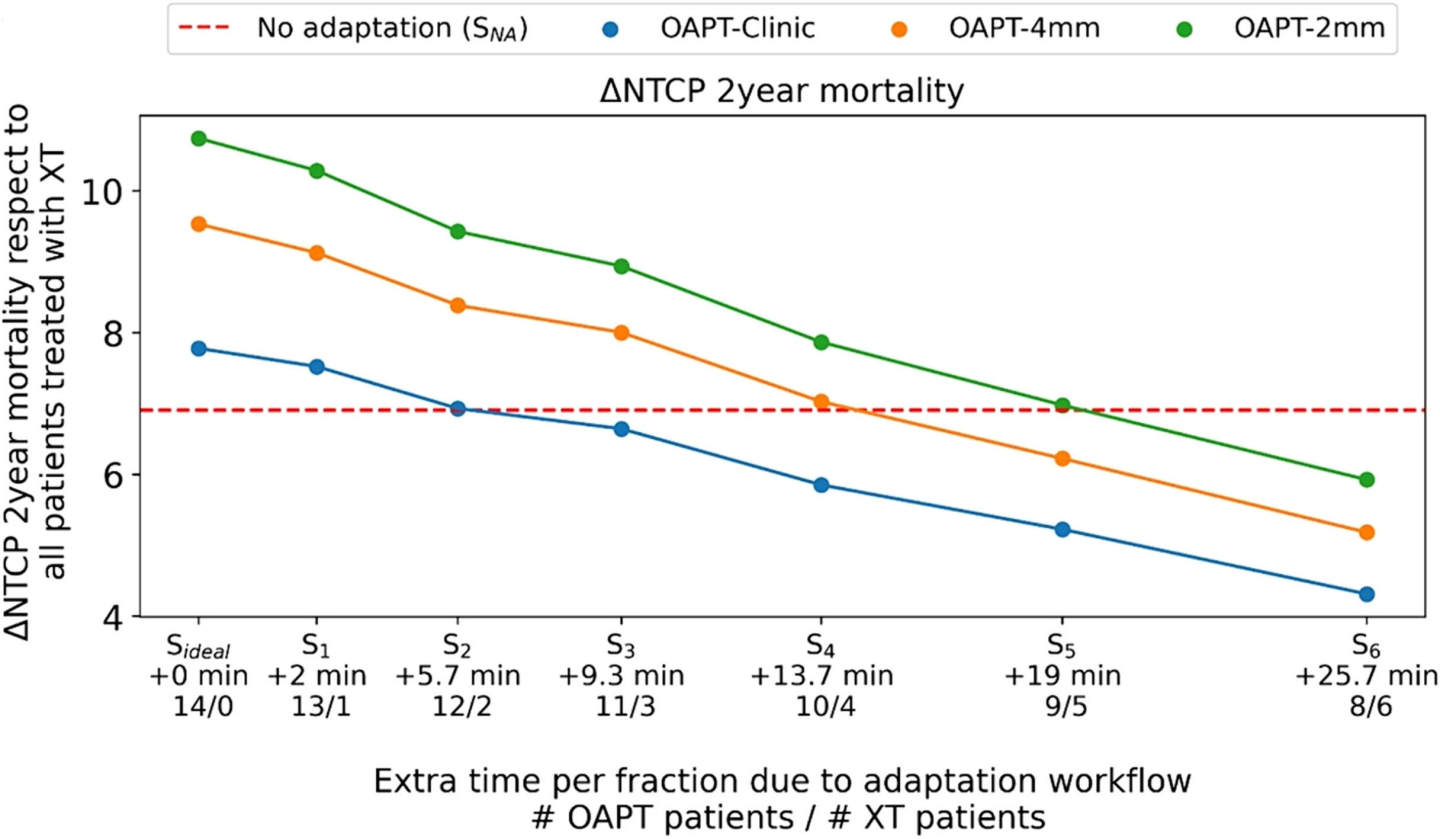
Variation in NTCP for 2-year mortality with three online adaptive proton therapy (OAPT) strategies using different setup margins for the lung cancer patient cohort investigated in (256) with institution-specific assumptions on facility occupancy and utilization. The dashed red line represents the NTCP gain for transitioning from conventional RT to non-adapted intensity-modulated PT. Points above this line indicate situations where OAPT strategies provide an NTCP gain across all patients. Seven different scenarios (S_ideal_, …,S_6_) are investigated, ranging from instantaneous OAPT for all patients (S_ideal_) to more realistic scenarios where an increasing patient proportion is treated with RT. For 2-year mortality, a maximum of 6 minutes of additional time per fraction per patient is allowed to maintain an overall NTCP gain without setup error reduction (blue line). With setup error reduction, 13.7 and 19 minutes of extra time per fraction can still provide an NTCP gain (orange and green lines). For details refer to (256). Reproduced from (256) with permission from Elsevier.

The clinical benefit and cost must be related with other aspects to get a full evaluation, like the potentially prolonged time of treatment and overall available time in the treatment room. However, OAPT could also enable hypofractionation thereby leading to an overall reduction of treatment time per patient. Novel studies exploring possible scenarios for the introduction of OAPT in PT centers with focus on clinical outcome and time expenditures have been recently published (256, 257) and further similar investigations are expected. Borderías et al. (256) specifically highlighted that when online adaptation is time-consuming and results in some patients being treated with XRT instead, there is a trade-off between clinical benefit (expressed as reduced normal tissue complication probability, NTCP, for the whole cohort) and the time available for adaptation (**Figure 5**). While the study results are highly illustrative, one should keep in mind that the general transferability to other institutions might be limited due to the made assumptions on facility utilization. According to the study, the benefit of online adaptation is contingent on reducing the setup margin, and a maximum allowable adaptation time can be defined, beyond which the modelled clinical advantage is no longer maintained. Furthermore, the availability of online adaptive strategies for PT with reduced setup margins would favor the employment of hypofractionation or dose escalation, which are currently limited by the treatment-induced toxicities. This has already been observed in XRT (258–261). Likewise, NTCP-based adaptation would also be facilitated (130), as well as emerging approaches like simulation-free workflows, which are gaining significant interest in XRT (262–264). In this sense NAPT bringing forward innovative adaptive strategies would thus be an invaluable tool for the introduction and exploration of novel therapies, enabling the treatment of previously inaccessible tumor entities.

In order to ensure a careful and nuanced balance between the benefits of OAPT treatments and the present limitations, it is important to consider each patient indication carefully. Factors such as the potential for hypofractionation or dose escalation enabled by OAPT, treatment time, margin reduction, clinical outcomes, available clinical resources, and the comparison between DAPT, NAPT, and offline adaptive workflows must all be weighed. It is crucial to recognize that there is no one-size-fits-all approach, and each patient’s specific circumstances should guide the choice of the most appropriate adaptive treatment strategy.

Thus, in light of the growing range of available workflows and adaptive strategies in PT and the interest in the topic of OAPT from both vendors and clinical users, further clinical implementations towards faster time regimes are soon to be expected, leading to a multifaceted technological landscape and approach, with decisions dependent on the specifics of each patient case and available workflow.
